# Reasons for leaving or remaining in nursing work and profession: a comparative analysis among emergency/urgent care professionals

**DOI:** 10.47626/1679-4435-2026-1514

**Published:** 2026-04-02

**Authors:** Maiara Bordignon, Emille Nair Lima dos Santos, Júlia Teixeira Ramos, Morgana Brandão, Veridiana Samuéla Alt, Inês Monteiro

**Affiliations:** 1 Universidade Federal da Fronteira Sul (UFFS), Curso de Graduação em Enfermagem, Programa de Pós-graduação em Enfermagem (PPGEnf), Chapecó, SC, Brazil; 2 Universidade Federal da Fronteira Sul (UFFS), Curso de Graduação em Enfermagem, Chapecó, SC, Brazil; 3 Universidade Estadual de Campinas (UNICAMP), Faculdade de Enfermagem, Campinas, SP, Brazil

**Keywords:** personnel turnover, job satisfaction, health personnel, emergency medical services, nursing., rotatividade de pessoal, satisfação no trabalho, profissionais de saúde, serviços médicos de emergência, enfermagem.

## Abstract

**Introduction:**

While the nursing workforce is fundamental to providing safe and quality access to health care, turnover poses a challenge to its stability.

**Objectives:**

To identify the reasons nursing professionals working in emergency/urgent care units intend to leave or stay in their current position, institution, or the nursing profession, determining the differences and/or similarities among these reasons by location.

**Methods:**

This exploratory, descriptive field study included 118 nursing professionals working in urgent care/emergency departments in 2 states in Brazil. The participants reported why they intended to remain in their current position, institution, and profession, or why they intended to leave, and the frequency of each reason was recorded.

**Results:**

Organizational factors related and working conditions were often cited as reasons for leaving. The reasons for staying included affinity with the work and the profession, coworkers, salary, and job stability. The reasons for intending to leave or remain in the current position, institution, and profession were diverse and varied among themselves. There were common factors and differences between the groups.

**Conclusions:**

Many of the reasons for intending leave were modifiable. Strategies to promote employee retention should include analysis of local factors.

## Abstract (pt)

**Introdução:**

Enquanto a força de trabalho em enfermagem é fundamental para o acesso à saúde de maneira segura e com qualidade, a rotatividade representa um desafio para a estabilidade dessa força de trabalho.

**Objetivos:**

Identificar os motivos pelos quais dois grupos de profissionais de enfermagem que atuavam em unidades de urgência/emergência tinham a intenção de deixar a unidade de trabalho, a instituição e a profissão na enfermagem e os motivos que os faziam querer permanecer; e analisar se há diferenças e/ou semelhanças nesses motivos entre os grupos por localidade.

**Métodos:**

Estudo de campo, exploratório e descritivo, com 118 profissionais de enfermagem que trabalhavam em unidades de urgência/emergência em duas cidades de estados diferentes do Brasil. Os profissionais relataram motivos geradores da intenção em permanecer na unidade de trabalho, instituição e profissão, assim como motivos de intenção em deixá-las, e a frequência foi contabilizada.

**Resultados:**

Fatores relacionados à organização e às condições de trabalho foram frequentemente citados como geradores de intenção em deixar. Os motivos de intenção em permanecer incluíram afinidade com o trabalho, equipe de trabalho, salário, estabilidade no emprego e afinidade com a profissão. Os motivos de intenção em deixar ou de permanecer na unidade, instituição e profissão foram diversos e variaram entre si. Houve fatores comuns e especificidades entre os grupos.

**Conclusões:**

Muitos fatores de intenção em deixar eram ajustáveis. As estratégias promotoras de retenção devem incluir a análise de fatores locais.

## INTRODUCTION

A 2025 World Health Organization report projected shortage of health care professionals in the coming years [^1^]. Although the global number of professionals has increased in the last decade, an estimated shortage of 11 million professionals is expected by 2030, in addition to a highly unequal distribution of nurses in different world regions [^1^]. The aging of the population and the nursing workforce, the growing demand for health services, and workload, management, and work structure issues are some of the factors associated with this shortage [^2^-^4^].

The training, recruitment, and retention of nursing professionals is a concern worldwide, since a lack of professionals with the required skills directly affects a country’s ability to meet its population’s health needs and can result in health insecurity [^1^,^2^,^5^,^6^]. Thus, researchers have consistently investigated factors that influence health workforce recruitment and retention [^7^]. Analyses of intention to leave or stay involve important indicators of workforce sustainability [^8^]. While the intention to stay predicts interest in remaining on the job or in the profession, intention to leave is a cognitive and conscious process in which workers consider leaving their position, their institution, and/or their profession in the near future [^8^].

From this perspective, it has been observed that some nursing professionals have a high intention to leave their jobs while others have a high intention to remain in their profession and current position. A greater understanding of these phenomena and associated factors has been the objective of several studies in recent years to provide necessary evidence for more effective human resource management for health care.

Brazilian studies have evaluated the intention to leave the profession and associated factors through instruments based on previously associated factors, such as workplace violence, job satisfaction, teamwork climate, a negative assessment of institutional support, and work overload [^9^-^11^]. However, other factors that contribute to intention to leave may not yet have been explored through specific measures. Thus, to broaden our understanding of these reasons, we investigated why nursing professionals intend to leave or stay in their current position and the profession. Our sample consisted of nursing professionals from urgent care/emergency departments in two cities in different states of Brazil: 1) to identify why these workers intended to leave of stay in their current position, institution, and the nursing profession; and 2) to determine the differences or similarities in these reasons according to location.

## METHODS

This exploratory descriptive field study included 118 nursing professionals (31 nurses and 87 nursing assistants or nursing technicians) who worked in urgent care/emergency departments (linked to the Unified Health System) in a city in Santa Catarina (n = 39) and a city in São Paulo (n = 79). The cities’ populations are estimated at 213,279 and 1,182,429 people, respectively [^12^]. The urgent care units were located in the state of Santa Catarina and the hospital emergency department (which is a referral center) was located in São Paulo.

This study, which is part of a larger research project on work ability, violence, and intention to leave among nursing professionals in emergency services, presents the results on these professionals’ intention to leave or stay in the current care unit, institution, or profession. The teams totaled 204 nursing professionals, of whom 130 participated in the study through random sampling [^9^], with 118 professionals responding to at least one of the semi-structured questions on the reasons they intend to leave or stay in the current care unit, institution, and profession.

The inclusion criteria were nursing professionals (nurses, nursing technicians, or nursing assistants) who had worked in the unit for at least 3 months. Workers in any shift or age group were eligible. However, those who were absent from work due to vacations or leaves during the data collection period were excluded, as were those who refused to respond to any of the questions on why they intended to leave or stay. These questions were part of a data collection instrument that included the features described below.

- Sociodemographic and work data were collected using the Questionnaire on sociodemographic data, lifestyle, health and work aspects [^13^].
- The Turnover Intention Scale [^14^], developed and validated in Brazil, includes 3 items on how much the participants think about, plan, or want to leave their institution in the future. The response options were: (1) never, (2) rarely, (3) sometimes, (4) often, and (5) always. Mean scores between 4 and 5 indicate a high intention to leave, scores between 3 and 3.9 indicate medium intention, and scores between 1 and 2.9 indicate low intention [^14^]. Cronbach’s alpha in this study was 0.92.
- Two questions assessed the professional’s intention to leave their current position and the profession: 1) Do you intend to stop working at the [unit name] unit, where you are currently located? 2) Do you intend to leave your profession? Responses ranged from 0 (no intention) to 10 (strong intention) [^15^].
- Three semi-structured questions investigated the 3 main reasons for the respondents’ intention to leave their current position, institution, and profession: 1) List the three main reasons why you intend to leave this unit; 2) List the three main reasons why you intend to leave this institution; and 3) List the three main reasons why you intend to leave this profession.
- Three semi-structured questions investigated the 3 main reasons for the respondents’ intention to remain in their current position, institution, and profession: 1) List the three main reasons why you have no intention of leaving this unit; 2) List the three main reasons why you have no intention of leaving this institution; and 3) List the three main reasons why you have no intention of leaving this profession.

Data were collected in person at each health unit by the first author. Participants individually completed the data collection instrument, delivering it to the researcher when completed, either on the same day or at a later date. Any questions the participants had about the study and/or instrument were addressed by the first author. Data were collected between 2016 and 2017, i.e., before the COVID-19 pandemic. The responses were entered into an Excel spreadsheet and were analyzed descriptively, counting the number of times each reason was cited. These reasons were grouped by similarity criteria, i.e., reasons with similar themes and meanings were grouped together, allowing a comparative analysis of the groups according to location (Santa Catarina vs São Paulo).

This study was approved by a Human Research Ethics Committee (Certificate 41225814.4.0000.5404). The privacy and anonymity of the participants were ensured.

## RESULTS

Of the 118 respondents, 66.9% worked in a hospital emergency department in São Paulo and 33.1% worked in urgent care units in Santa Catarina. Table 1 provides further information on each group.

**Table 1:** Sociodemographic and work characteristics (n = 118)

Variables	Santa Catarina n = 39 n (%) or mean (min.-max.)	São Paulo n = 79 n (%) or mean (min.-max.)
Professional category		
Nurse	10 (25.6)	21 (26.6)
Nursing assistant and/or technician	29 (74.4)	58 (73.4)
Sex		
Female	35 (89.7)	63 (79.7)
Male	4 (10.3)	16 (20.3)
Conjugal status		
Without a partner	23 (59.0)	35 (44.3)
With a partner	16 (41.0)	44 (55.7)
Children		
No	10 (25.6)	24 (30.4)
Yes	29 (74.4)	55 (69.6)
Education		
Technical school, complete	18 (47.4)	30 (38.5)
University, incomplete	8 (21.1)	12 (15.4)
University, complete	5 (13.2)	21 (26.9)
Graduate	7 (18.4)	15 (19.2)
No response (Santa Catarina: 1; São Paulo: 1)		
Employment type		
Civil servant (stable public employment)	35 (89.7)	44 (60.3)
Temporary, indeterminate time (CLT)		23 (31.5)
Temporary, set time	4 (10.3)	2 (2.7)
Other		4 (5.5)
No response (São Paulo: 6)		
Work shift		
Morning	6 (17.1)	21 (28.4)
Afternoon	7 (20.0)	23 (31.1)
Night	17 (48.6)	28 (37.8)
Morning and afternoon	5 (14.3)	2 (2.7)
No response (Santa Catarina: 4; São Paulo: 5)		
Age (years)		
No response (Santa Catarina: 1; São Paulo: 3)	37.6 (23-60)	37.3 (22-68)
Time worked in the unit (years)	2.4 (4 months to 8 years)	4.6 (3 months to 26 years)
Time worked in the institution (years)		
No response (São Paulo: 2)	5.4 (4 months to 24 years)	6.2 (3 months to 27 years)
Intention to leave the unit (0-10)^*^	2.8 (0-10)	2.8 (0-10)
Intention to leave the institution (1-5) ^†^		
No response (Santa Catarina: 1; São Paulo: 3)	1.7 (1-5)	1.6 (1-4.3)
Intention to leave the profession (0-10)^*^	1.4 (0-10)	1.7 (0-10)

* Scale range: between 0 (no intention) e 10 (strong intention).

† Scale range: between 1 (never) and 5 (always).

The main reasons for intending to leave the hospital emergency department (São Paulo) were work overload, physical and mental health issues, overcrowding or an excess of patients, unit infrastructure, and work hours. The main reasons for intending to leave the urgent care units (Santa Catarina) were work hours (e.g., working weekends/holidays), health and salary issues, high demand for care, and long commuting distance. In both groups, salary and a lack of recognition were among the most cited reasons for intending to leave both the institution and the profession (Table 2).

**Table 2:** Most frequently cited reasons for intending to leave the current position, institution, and profession by location

	São Paulo (times reported)	Santa Catarina (times reported)
Intention to leave the urgent care/emergency department. ^*^	- Work overload (14); - Physical or mental health (physical exertion, fatigue, stress) (13); - Overcrowding or excess of patients (12); - Sector infrastructure (5); - Distribution of working hours (the fact of working on weekends or the desire to work 30 hours a week) (5); - New knowledge or greater learning opportunities in another area or sector (5); - Change of professional category or area (4); - Lack of security (4); - Lack of appreciation, respect or professional recognition (4); - Retirement (2); - Unhealthy working conditions (2); - Opportunity for professional growth (2); - Coworkers (2); - Lack of training or professional qualification opportunities (2); - Feeling of not having provided the desired assistance (2); - Stable employment with better working conditions or remuneration (2);	- Distribution of work hours/schedule (working weekends, nights, etc.) (14); - Physical or mental health (health, fatigue, stress) (3); - Salary (3); - High patient flow and turnover (demand for care) (3); - Distance between home and work (3); - Lack of security (3); - Interpersonal relationships (conflicts, relationships with colleagues or with leadership) (3); - Change of work environment (2); - Exposure as a health care professional (vulnerability) (2).
Intention to leave the institution ^†^	- Salary (6); - Lack of professional appreciation and recognition (5); - Change of area, another job opening or new professional qualification (5); - Physical, mental health and/or quality of life (5); - High workload or overcrowding (5); - Better working conditions (safer, less stressful work environment) (4); - Long commute distance (3); - Job security (3); - Retirement (2); - Schedules (working weekends/holidays) (2); - Management (perceived neglect or lack of connection between management and the care team) (2).	- Lack of professional appreciation and recognition (5); - Salary (3); - Lack of security and vulnerability to violence (2); - Another job opening or another job opening closer to family (2); - Hours and work on weekends (2).
Intention to leave the profession ^‡^	- Lack of professional appreciation and recognition (9); - Salary (7); - Health (stress, anxiety, fatigue, etc.) (5); - Change of professional category or area (3); - Work conditions (3); - Lack of respect for professionals (2); - Workload (2); - Retirement (2); - Schedule and work on weekends and special dates (2); - Relationship between colleagues (disunity and/or competitiveness) (2).	- Salary (4); - Lack of professional appreciation and recognition (3); - New profession (2).

* São Paulo: 45 participants answered the question; Santa Catarina: 22 participants answered the question.

† São Paulo: 30 participants answered the question; Santa Catarina: 11 participants answered the question.

‡ São Paulo: 30 participants answered the question; Santa Catarina: 10 participants answered the question.

The 3 most cited reasons for intending to remain in the emergency department (São Paulo) were: coworkers, affinity for the work, and the relationship with leaders/supervisors. The 3 most cited reasons for wanting to remain in the urgent care units (Santa Catarina) were: affinity for the work, flexible work hours, and coworkers. For both groups, the main reasons for intending to remain at the institution were salary issues and job stability. Affinity with the profession, enjoying the work, personal and professional satisfaction and fulfillment were the most cited reasons for intending to remain in the profession (Table 3).

**Table 3:** Most cited reasons for intending to remain in the current position, institution, and profession among professional groups by location

	São Paulo (times reported)	Santa Catarina (times reported)
Intention to remain in the urgent care/emergency department ^*^	- Coworkers (relationship between colleagues and with the multidisciplinary team)/“I like the people I work with” (37); - Enjoyment of working in urgent/emergency care/“I like what I do” (30); - Leadership/supervisor (15); - Dynamism of the sector (e.g., characteristics of several sectors in one, rotation of activities) (11); - Opportunity for new knowledge and participation in projects (11); - Affinity/love for the profession (6); - Enjoyment of the work environment (6); - Schedules, days off or 30-hour work week (5); - Opportunity to help/care for other people and protect life (5); - Family or personal needs (5); - Job stability (4); - Resources offered by the unit, working conditions or expectation of improvement in working conditions (4); - Sector routine (3); - Salary (3); - Feeling recognized and respected as a professional (e.g., praise) (3); - Choice as a professional career: “I chose to work here” or “this unit” (3); - Affinity for the patients (pediatrics) (3); - Awaiting retirement (2); - Personal satisfaction (2). - Being accustomed to the work or sector (2); - Job satisfaction (2).	- Enjoyment of urgent/emergency care work: “I like what I’m doing,” “I like working in this unit” (18); - Work schedule (flexibility) (11); - Coworkers (11); - Good work environment (5); - Relationship with leadership (5); - Job stability (4); - Salary (3); - Short commute distance (3); - Enjoyment of the profession (2); - Opportunity for overtime and extra income (2); - Learning opportunity (2).
Intention to remain at the institution^†^	- Salary (28); - Opportunity to acquire knowledge, learning and/or professional growth (24); - Job stability (23); - Work environment: “good place to work”/“I am satisfied with my workplace” (10); - Benefits (9); - Teaching hospital (8); - Workload (30 hours per week), work schedule, days off (7); - Coworkers (8); - Job satisfaction (7); - Relationship with leadership (5); - Recognition the institution has in relation to other institutions (4); - Policy of valuing and respecting professionals (4); - Continuing to practice the profession (3); - Environment with resources and materials for work (3); - Work conditions (3); - Autonomy (3); - Work dynamics in the institution (2); - Possibility of caring for other people (2); - Hope for improvement (2); - Professional desire and choice (2); - Career and salary plan (2).	- Job stability (18); - Salary (12); - Flexible hours (8); - Enjoyment of the work (6); - Coworkers (6); - Work environment (3); - Opportunity to acquire knowledge and experience (2); - Continue in the profession (2); - Professional recognition (2); - Proximity to family (2); - Enjoyment of working at the institution (2); - Good work conditions (2).
Intention to remain in the profession^‡^	- Enjoy/love/ passion for the profession: “I can’t imagine doing anything else in life”/“a profession that I like” (37); - Enjoyment of the work: “I like what I do” (18); - Personal and professional satisfaction/fulfillment (16); - Patient care, opportunity to help people or provide assistance during difficult times (16); - Salary or prospect of a raise (8); - Marketable job skill: “profession with good acceptance and scope in the job market” (7); - Knowledge and learning for career, life and/or to improve as a human being (6); - Work hours (30 hours per week) (5); - Interaction with other people (3); - Vocation (2); - Awaiting retirement (2); - Coworkers (2); - Family (2); - Professional and personal reward (2).	- Enjoy/love the profession: “I can’t see myself in another profession” (15); - Enjoyment of the workplace or the profession: “I like what I do” (11); - Professional and personal satisfaction/fulfillment (5); - Professional appreciation and recognition (4); - Years of service or age (4); - Enjoyment of the health care field (2); - Flexible hours (2); - Interaction with other people (2); - Coworkers (2); - Salary (2); - Patient well-being and care (2).

* São Paulo: 71 participants answered the question. Santa Catarina: 29 participants answered the question.

† São Paulo: 69 participants answered the question. Santa Catarina: 31 participants answered the question.

‡ São Paulo: 69 participants answered the question. Santa Catarina: 29 participants answered the question.

## DISCUSSION

The sample predominantly consisted of nursing assistants or nursing technicians and women, which aligns with the characteristics of Brazil’s nursing workforce, as described in another study [^16^]. Our sample was relatively young, with most being < 40 years of age. Nevertheless, their age ranged from 22 to 68 years, which allowed analysis of intention to leave or remain in the current position and/or profession at different life and career stages.

This study’s focus was to identify why nursing professionals intended to leave or stay in their current position, institution, or profession. The reasons they reported were diverse, both for intending to leave and intending to stay, demonstrating that, in general, no single reason leads nurses to leave their current position or the profession [^7^].

Regarding the intention to leave the current position, 16 different reasons were reported > 1 time by the São Paulo group, and 9 different reasons were reported > 1 time by the Santa Catarina group. Work-related factors were the most cited reasons for intending to leave the unit in both groups. Although the groups differed in both the reasons and reporting frequency, work overload, unit overcrowding, high demand for service, physical and mental health, unit infrastructure, workload issues (such as working on weekends, holidays, or special dates) and salary issues were all reported. Regarding the intention to leave the institution and/or the profession, salary issues and lack of professional appreciation and/or recognition were the most cited reasons in both groups.

Through interviews with nursing managers in Macau, China, a study assessed factors contributing to the shortage of nurses [^2^]. Their results corroborate ours, highlighting the need for better management of workloads, and communication about salary and benefits, staff availability, and continuing education [^2^]. U.S. hospital nurses of different generations reported workload as a reason for intending to leave their current position [^17^]. Aging-related challenges, such as a physically demanding workload, have also been reported as a reason for quitting among older nurses [^17^].

A Swedish study of 2,474 nurses explored the factors associated with intention to leave the profession, and turnover among experienced nurses (i.e., those who graduated 11, 13, and 15 years ago) [^7^]. Those considering leaving the profession reported factors such as workload, lack of organizational resources, non-compliance with nursing values, lack of recognition, deteriorating health, unfair salary in relation to workload, commitment, schedules, or too great an impact on personal and family life [^7^].

Our results further validate the evidence of other studies on the reasons nurses intend to leave their current position. Workplace violence, perceived weaknesses in nursing care provision, health issues, and high workload are examples of factors described in previous studies [^9^,^18^-^21^], as well as our own. This combined evidence suggests that initiatives to promote health, workplace safety, better organization of work processes, schedules, and reward strategies, in addition to initiatives to guarantee quality care, can lead to greater retention of the health workforce.

However, although there were common factors between the groups regarding intent to leave their current position or the profession, specific workplace factors should be considered, in addition to life factors. These aspects shape professional experience and tend to influence the desire to leave or stay in the current position or institution.

A study of nurses from 78 Belgian hospitals with intensive care units investigated the prevalence of intention to leave the current position or the profession and these variables’ relationship with the work environment [^22^]. Two years after the COVID-19 pandemic, there was a high prevalence of intention to leave, although there were differences between hospitals, which might be explained by aspects specific to the work environment [^22^]. Intention to leave the current position or the profession was lower in hospitals with better perceived work environments [^22^].

Promoting better work environments can involve several factors, including: 1) involving professionals in institutional matters; 2) providing adequate personnel and resources; 3) adhering to the fundamentals of quality nursing care; 4) increasing leadership and support capacity among nursing managers; and 5) improving relationships between nurses and physicians [^22^,^23^]. Thus, the literature indicates that building favorable work environments, reducing workload, and promoting salaries are some areas of intervention that can attract and retain nursing professionals and achieve better patient outcomes [^24^,^25^]. In the present study, salary, learning and professional growth opportunities, job stability, and the work environment were also among the most cited reasons for intending to remain at the institution, as has been reported in previous studies [^22^,^24^,^26^].

The relationship quality between workers and leadership was also an important factor in the intention to remain in the current position, as were affinity with urgent/emergency care work, unit dynamics, learning and professional growth opportunities, scheduling, and a good work environment. Among those who intended to stay in their current position, good relations with leadership were interpreted as having leaders who are open to dialog, concerned about the individual needs of professionals, trustworthy, offer support when necessary, and recognize the work done.

A study on Canadian home care professionals identified strategies that could contribute to greater worker retention at the agency, finding that higher salaries were foremost [^6^]. Love for and commitment to clients and the emotional bond with coworkers also explained worker retention at the agency [^6^].

Other studies have reported that the relationship between workers and leadership is influential in worker retention [^27^-^29^]. Studies have found that leadership behavior is a predictor of intention to remain in the current position [^28^,^29^], indicating that it is up to nursing managers to provide continuing education and professional development, thus improving leadership skills among nurses [^27^ ^29^]. Such initiatives can promote effective leadership, address retention issues in care units, improve organizational culture, and strengthen nursing care [^27^-^29^].

In the present study, the most cited reasons for intending to remain in the profession were affinity for the profession, enjoying the work, professional and personal satisfaction, patient care, and the opportunity to help people or provide assistance during difficult moments. As shown in a previous study, these factors can also be important motivators for nurses [^30^]. That study, which included nurses working in health services in Brazil, found that the act of caring itself was a source of motivation [^30^]. The results also indicated that professional recognition, institutional support, and improved working conditions can motivate nurses in their profession [^30^].

Our inclusion of professionals of different ages and years of service showed that some initiatives for worker retention can be applied to all nursing professionals, regardless of years at the institution [^17^], given that certain reasons for wanting to leave (e.g., lack of recognition and poor working conditions) or to stay (e.g., salary and job security) were commonly reported across the sample.

Although our data were collected between 2016 and 2017, the topic of this study is still under discussion in literature and continues to be a global challenge for health systems [^1^]. Our results corroborate evidence from recent research, including studies conducted in other countries, thus advancing our understanding nurses’ reasons for intending to leave or remain in their current position or the profession in Brazil. These data can be an important resource for health care human resource management and can be used in longitudinal monitoring of worker retention. New studies on the topic can be conducted in Brazil, including professionals from other states and institutions, as well as nurses not involved in urgent/emergency care. This could help minimize the limitations of our study, which include the fact that only 2 institutions were represented, making it difficult to generalize the results. Our results also indicate that, although there are common factors among groups of professionals working in different regions, site-specific factors may reflect the characteristics of each workplace and warrant individual analysis. By applying validated scales and other analytical methods, other studies could also assess the impact of the factors described in this study as predictors of intention to leave or remain in the current position and the profession.

## CONCLUSIONS

The reasons nurses reported for intending to stay in their position, institution, and profession were diverse and included affinity for the work and the profession, the relationship with coworkers, salary, and job stability. The reasons for intending to leave included organizational issues and work conditions, such as work overload, lack of recognition, salary, and scheduling. Comparative analysis between the groups revealed similarities among the reasons, although the groups differed regarding workplace factors, which should be further investigated to develop more focused initiatives. The reasons for intending to leave or stay in the current position, institution, and profession varied, i.e., the reasons for intending to leave the unit may not be the same as those for intending to leave the institution. Thus, strategies to increase retention should be based on continuing analysis of local factors by team leaders and management. Many of the reasons given for intending to leave were modifiable, and thus, avoidable factors.

## Data Availability

The data cannot be made publicly available due to ethical reasons
